# Topical Application of SVF/PRF in Thermal Injuries—A Retrospective Analysis

**DOI:** 10.3390/jcm14134710

**Published:** 2025-07-03

**Authors:** Lukas Naef, Mauro Vasella, Jennifer Watson, Gregory Reid, Tabea Breckwoldt, Matthias Waldner, Luzie Hofmann, Michael-Alexander Pais, Philipp Buehler, Jan Alexander Plock, Bong-Sung Kim

**Affiliations:** 1 Department of Plastic Surgery and Hand Surgery, University Hospital Zurich, 8091 Zurich, Switzerland; lukas.naef@usz.ch (L.N.); mauro.vasella@usz.ch (M.V.); luzie.hofmann@usz.ch (L.H.); 2 Department of Plastic Surgery and Hand Surgery, Cantonal Hospital of Frauenfeld, 8501 Frauenfeld, Switzerland; 3 Department of Intensive Care Medicine, Cantonal Hospital of Winterthur, 8401 Winterthur, Switzerland; 4 Department of Plastic Surgery and Hand Surgery, Cantonal Hospital of Aarau, 5001 Aarau, Switzerland

**Keywords:** burn, stem cell, SVF, PRF

## Abstract

**Background**: The traditional management of acute burn wounds using eschar debridement followed by split-thickness skin grafting has notable drawbacks. Stromal vascular fraction (SVF), derived from autologous adipose tissue, promotes epithelialization and angiogenesis, while platelet-rich fibrin (PRF), obtained via centrifugation of patient blood, enhances wound healing. This study retrospectively analyzes the outcomes of patients with thermal injuries treated with a combination of topical SVF and PRF at the University Hospital Zurich Burn Center. **Methods**: From 2018 to 2020, 13 patients with deep partial-thickness burns (DPTBs) or mixed-pattern burns (MPBs) received combined topical SVF and PRF treatment. Eschar removal was performed enzymatically or surgically following hydrotherapy. SVF was collected via liposuction, and PRF from centrifuged blood. Healing progress, additional surgeries, and scar outcomes (assessed by the Manchester Scar Scale, MSS) were evaluated retrospectively. **Results**: The mean total body surface area burned was 29.6%, with 6.3% treated using SVF and PRF. Five patients required further surgical intervention for residual defects. Complete healing occurred within 20 days in patients without residual defects and within 51 days in those with defects. Higher MSS scores were observed in patients requiring additional surgery. No adverse effects were noted. **Conclusions**: Topical SVF and PRF offer a potentially less-invasive treatment for MPB and DPTB. However, due to frequent residual defects and regulatory concerns around SVF use, this approach cannot yet be considered a standard treatment.

## 1. Introduction

Despite ongoing research, excision followed by coverage by split-thickness skin grafts (STSGs) remains the gold standard for the treatment of mixed-pattern burns (MPBs), deep partial-thickness burns (DPTBs), and full-thickness burns (FTBs) [1]. Early debridement is recommended and is most commonly carried out either surgically or enzymatically using a bromelain-based agent [2]. Following the removal of eschar, MPBs, DPTBs, and FTBs require appropriate coverage as conservative treatment is associated with delayed wound healing, hypertrophic scarring, contraction, and infections [3]. Despite their undoubtable benefits, STSGs are accompanied by disadvantages such as limited availability in severely burned patients, blood loss, donor site morbidity, and secondary contractures [4].

Novel technologies and the modification of already established methods try to challenge the disadvantages of STSGs [5]. In this context, regenerative plastic surgery has established itself as an auspicious concept that relies on autologous reparative resources. The rise of mesenchymal stromal cells (MSC) and the facile harvest of adipose-derived stromal cells (ADSC) from subcutaneous fat depots have further nurtured the hope for less-invasive solutions in wound coverage [6]. ADSCs have been reported to promote epithelialization, angiogenesis, and immunomodulation [7,8]. Nevertheless, the time needed for the cultivation of ADSCs may lead to contamination and is impractical for single-stage surgeries, which highlights the importance of using uncultured progenitor cells [9].

The stromal vascular fraction (SVF), a heterogenous cell mixture that is routinely isolated by enzymatic digestion or mechanical isolation of adipose tissue, contains a significant amount of regenerative progenitor cells including ADSCs, and has been shown to have antioxidant, antiapoptotic, and immune-modulatory effects [10,11,12,13]. Platelet-rich fibrin (PRF), which is harvested by the centrifugation of autologous patient blood, contains significant amounts of platelets, growth factors, and cytokines trapped in a fibrin matrix [14]. Several preclinical and clinical studies reported a positive course of wound healing associated with the use of PRF in soft tissue defects, showing benefits such as improved wound angiogenesis and connective tissue formation [15,16,17].

Beyond the sparse preclinical studies and case reports, there is limited clinical data regarding the utility of topical SVF application with or without PRF supplementation in the treatment of acute burn wounds in humans. In the presented case series, we conducted a descriptive retrospective evaluation of 13 patients with thermal injuries that were treated by a topically applied combination of SVF and PRF at the Burn Center of the University Hospital Zurich.

## 2. Material and Methods

### 2.1. Patient Selection

The data of all patients with DPTBs and MPBs that were topically treated with a combination of SVF and PRF at the University Hospital Zurich between 2018 and 2020 was retrospectively analyzed. Exclusion criteria were superficial-thickness burns, superficial partial-thickness burns (SPTBs), and FTBs. Burned TBSA and depth were defined on admission by the attending burn surgeon in accordance with standard guidelines [18]. The indication for SVF and PRF application was made by the attending burn surgeons. Whenever possible, patients were followed up until complete wound closure. This study was approved by the local ethics committee (BASEC-Nr. 2024-01978). Consent for study participation and permission for the publication of anonymized clinical images were signed by the patients.

### 2.2. Debridement, Production, and Application of SVF and PRF

On admission, all patients underwent hydrotherapy for initial wound debridement and the removal of blisters. Next, either a surgical excision or enzymatic debridement with Nexobrid^®^ (Mediwound, Yavne, Israel) was carried out. The enzymatic debridement was performed after a pre-soaking phase of at least 4 h using Prontosan Wound Gel^®^ (B. Braun, Melsungen, Germany). The post-soaking phase consisted of Prontosan^®^-soaked gauzes for an additional 12–18 h. Following careful inspection, an experienced burn surgeon provided the indication for successive treatment with SVF and PRF.

The SVF was harvested from uninjured subcutaneous adipose tissue depots by liposuction after infiltrating a tumescent solution of ringer lactate with 0.2% adrenaline. The lipoaspirate was then processed according to the protocol of the GID SVF-2 System (GID BIO, Louisville, CO, USA), a sterile closed disposable cellular isolation device designed for isolating SVF from lipoaspirates [19]. Within this system, the lipoaspirate was washed three times with 100–125 mL ringer lactate (36–39 °C). The lipoaspirate was then disaggregated using the GIDZyme-2, a collagenase enzyme for ex-vivo dissociation of human tissue to release nucleated cells from the extracellular tissue matrix, within an incubator for 45 min at 41 °C. The final SVF was then isolated by two centrifugation (600× *g*/min) steps.

PRF was obtained by drawing autologous venous blood (approximately 120 mL) at the beginning of the surgery which was mixed with citrate and transferred together into the Vivostat^®^ system (Vivostat, Borupvang, Denmark). The Vivostat^®^ system offers a fully automated process that generates autologous PRF.

The final SVF as well as PRF were therefore transferred into one of the two slots of the Vivostat^®^ co-delivery applicator unit. The mixture of SVF and PRF was then topically applied to the wounds with the Vivostat^®^ Spraypen. The wounds were covered with Mepitel^®^ (Mölnlycke, Gothenburg, Sweden), a silicone-based dressing, and dry gauze. The whole process of SVF and PRF isolation was performed within the same surgery as the debridement of the treated area.

In three cases (patient #3/7/13) presenting DPTB or even FTB, STSGs were applied in addition to SVF and PRF in the same surgery. Wounds were covered with Bactigras^®^ (Smith & Nephew, London, UK) and sterile gauze. The first dressing change was performed five days after surgery. If the wounds did not show proper healing, another surgical debridement followed by STSG transplantation was performed.

### 2.3. Outcome Assessment

**Figure 1:**
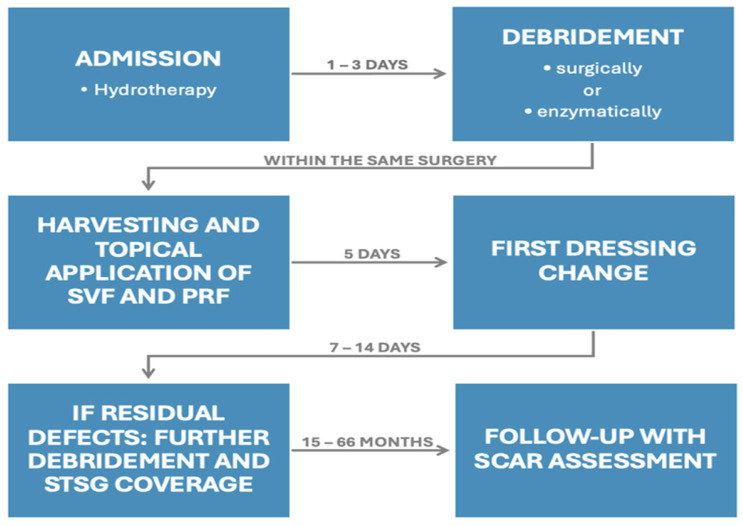
Schematic of the therapeutic process and its key timepoints.

Wounds were photo-documented on the day of admission, post-debridement, at the first dressing change on the fifth post-operative day, and in the outpatient follow-up consultations. TBSA as well as the burn degree of the wound treated with SVF and PRF were evaluated. Also, the following time intervals were examined: trauma to debridement, debridement to SVF therapy, SVF therapy to treatment of residual defects, time-to-heal, and hospitalization. A special interest was to analyze whether wounds fully healed under the SVF and PRF treatment or if residual defects remained that demanded further surgeries. Complete wound healing was considered as the presence of clinical epithelization of the entire treated area. Finally, the Manchester scar scale (MSS) was used to rate scarring and was assessed by five Board-certified plastic surgeons. The score is based on the visual analog scale (VAS) of color, finish, contour, distortion, and texture, and ranges from a minimum score of 4 to a maximum of 14, being the worst possible score [20]. Data is represented as range and mean ± SD. Figures were created with GraphPad Prism V8.0 (GraphPad Software, San Diego, CA, USA) (Figure 1).

## 3. Results

### 3.1. Patients

A total of 13 patients (7 male, 6 female) were included in this study. The mean age was 45.3 years (range 28–68 years; SD ± 13.58). The mechanism of injuries included flames in nine patients, while the other four patients suffered from scalds. The mean TBSA was 29.6% (range 6–85%; SD ± 25.82). More specifically, the mean body surface area (BSA) of SPTB was 7% (range 2–16%; SD ± 4.61), DPTB 12.8% (range 2–46%; SD ± 13.02), and FTB 20% (range 2.5–50%; SD ± 18.18). None of the patients suffered from an inhalation injury and the average abbreviated burn severity index (ABSI) was 6.54 (range 3–13; SD ± 3.5).

### 3.2. Debridement and Treatment with SVF and PRF

The mean BSA treated with SVF and PRF was 6.3% (range 1–15%; SD ± 4.7), mostly localized on the upper extremities, especially the hands but also in the face, abdomen, thighs, or gluteal area. Among the thirteen patients, five patients (patient #1/4/7/12/13) received the SVF and PRF therapy only for DPTBs, whereas in seven patients (patient #2/5/6/8/9/10/11), MPBs that included the combination of SPTBs and DPTBs were treated with SVF and PRF. Only one patient (patient #3) received the SVF and PRF combination for MPB that included DPTBs and FTBs. The first debridement occurred on average within 1.6 days (range 1–3 d; SD ± 0.7) post-trauma and was performed enzymatically with Nexobrid^®^ in nine cases (patient #1/4/5/6/7/8/9/11/12), while the other four patients received a surgical debridement (Table 1). Among the four patients undergoing surgical excision, three (patient #2/10/13) received tangential excision. In patient #3, where DPTBs were mixed with FTBs, an epifascial excision was performed. The elapsed time between the first debridement and the coverage with SVF and PRF was on average 2.4 days (range 0–6 d; SD ± 2.1). In three patients, the combination of SVF and PRF was applied in conjunction with STSG for wounds presenting DPTBs only (patient #7/13) or wounds where DPTBs were mixed with FTBs (patient #3). The remaining 10 patients were treated exclusively with the combination of SVF and PRF.

### 3.3. Residual Defects and Time-to-Heal

Residual defects that required further surgical interventions occurred in five (patient #1/2/7/8/10) of the thirteen cases. The mean BSA of the residual defects was 6.6% (range 2–15%; SD ± 6.0) and occurred in different locations such as the face, hands, gluteal area, or abdomen. In all those five cases, the residual defects presented almost in the entire area that was initially treated with SVF and PRF. One of the five cases (patient #7) presented residual defects despite the fact that SVF and PRF were combined with STSG initially. Further treatment of residual defects was performed with STSG within 20 days (range 11–34 d; SD ± 9.6) on average after the initial treatment with SVF and PRF. Complete epithelization of the entire treated area was reached, on average, within 33 days (range 10–50 d; SD ± 17.7) across all cases. The cases with surgically treated residual defects increased the average time-to-heal in general as they healed on average within 51 days (range 20–60 d; SD ± 8.8). It is noteworthy that the time-to-heal from the coverage of residual defects with STSG to complete epithelization was 13.4 days (range 9–21 d; SD ± 4.6), which is noticeably shorter than the time from SVF and PRF application to complete epithelialization in the group without residual defects, which was on average 20 days (range 14–28 d; SD ± 7.5). Specification of the cases with residual defects and time-to-heal is shown in Table 2. Patients #12 and #13 died within 11 and 19 days after admission with missing outcome analyses. Patient #12, with an ABSI score of 13, succumbed to multiple organ failure precipitated by a fulminant septic shock due to multidrug-resistant pneumonia with acute respiratory distress syndrome. Patient #13, with an ABSI score of 11, also died due to multiple organ failure after a fulminant septic shock due to multidrug-resistant pneumonia and endocarditis. In both cases, no correlation between their deaths and the therapy with SVF and PRF could be observed.

### 3.4. Hospitalization Time

The mean hospitalization time was 38 days (range 10–111 d; SD ± 30.1) within all cases. The mean hospitalization time of patients with residual defects was 56 days (range 21–111 d; SD ± 36.2), whereas the mean hospitalization time of the group not showing residual defects of the area treated with SVF and PRF was 24 days (range 12–39 d; SD ± 11.5). Eight patients (patient #1/2/3/4/6/7/8/10) were treated in the intensive care unit for an average of 26 days (range 2–76 d; SD ± 26.1).

### 3.5. Aesthetic Outcome

**Figure 2:**
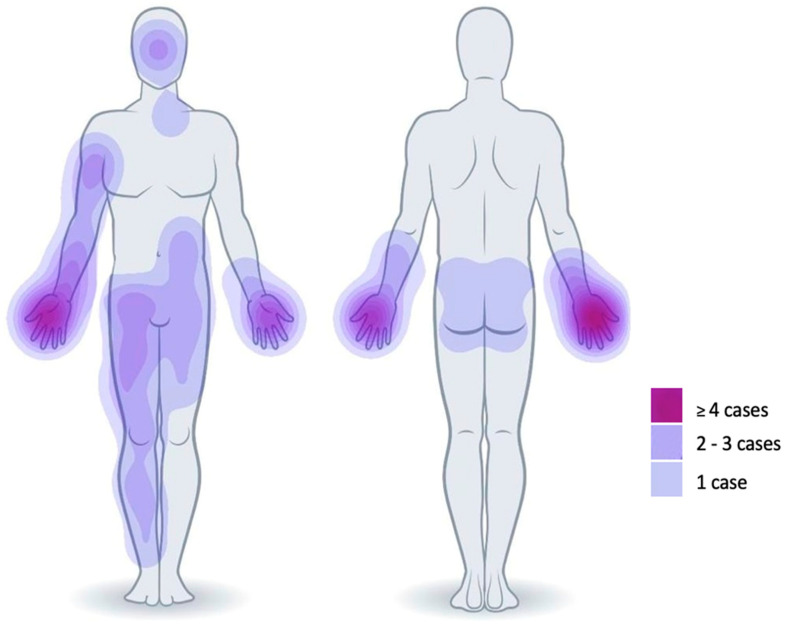
Heatmap illustrating the anatomical regions treated with SVF and PRF. The primary areas of treatment were the hands, with additional treatments administered to the face, trunk, gluteal region, and lower extremities.

**Figure 3:**
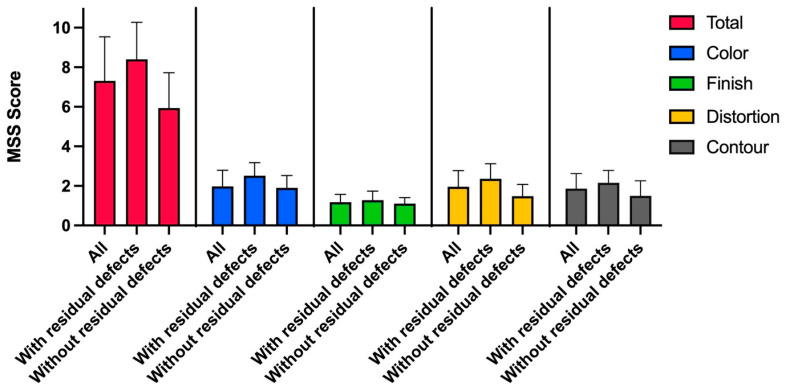
Mean total MSS score as well as for color, finish, contour, and distortion of all patients and of the cases with and without residual defects specifically.

**Figure 4:**
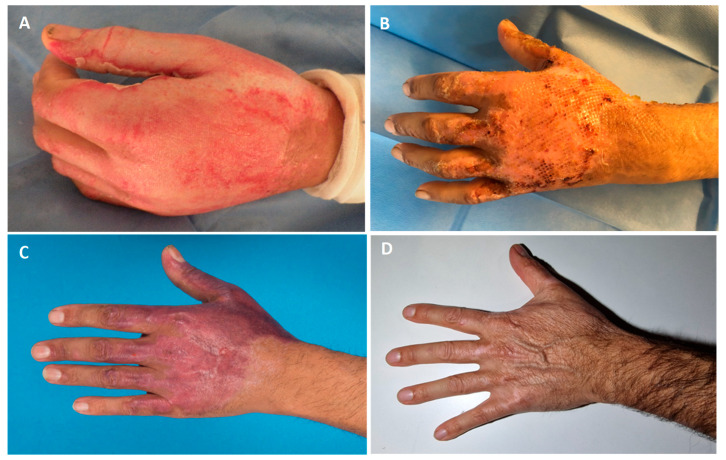
Patient #5: 42-year-old male patient with MPBs of the left hand receiving the combination of SVF and PRF 4 days after the enzymatic debridement. (**A**) After the enzymatic debridement with bromelain; (**B**) 5 days after SVF and PRF application; (**C**) 21 days after SVF and PRF application; (**D**) final result after 5 years.

**Figure 5:**
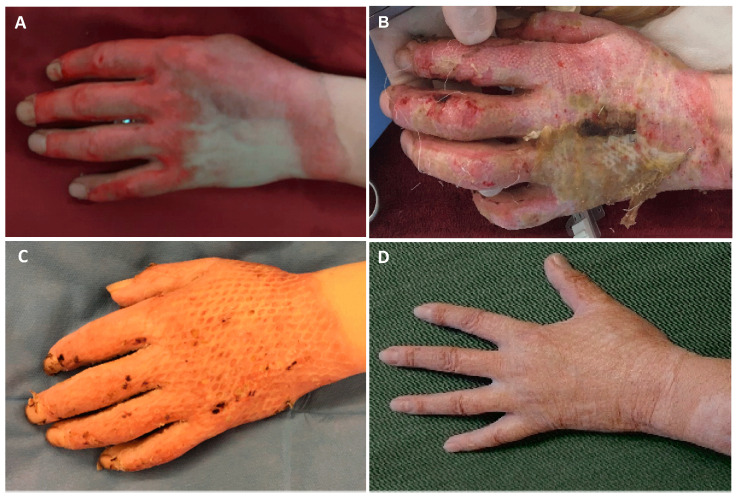
Patient #1: 41-year-old male patient with MPBs of the left hand receiving the combination of SVF and PRF 3 days after the enzymatic debridement with bromelain. (**A**) Before the enzymatic debridement with bromelain; (**B**) 14 days after SVF and PRF application. Due to residual defects, STSG covering was performed (**C**) 14 days after STSG application and 28 days after initial SVF and PRF treatment. (**D**) Final result 5.5 years after STSG application.

**Figure 6:**
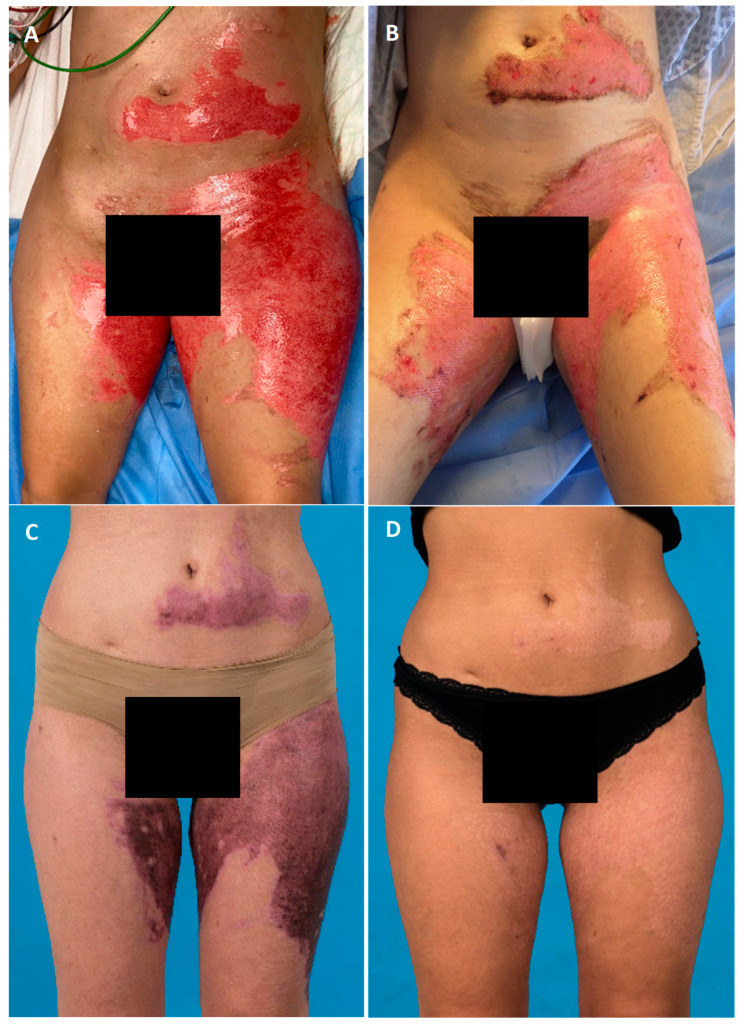
Patient #11: 28-year-old female patient with MPBs of thighs and abdomen receiving the combination of SVF and PRF 4 days after the enzymatic debridement. (**A**) After enzymatic debridement with bromelain; (**B**) 5 days after SVF and PRF application; (**C**) 2 months after SVF and PRF application; (**D**) final result 2.5 years after SVF and PRF application.

The median follow-up was 35 months (range 15–66 months; SD ± 17.6) after trauma. Follow-ups of patients #12 and #13 were missing due to their respective deaths at 11 and 19 days after admission. Furthermore, patients #3 and #6 did not present for follow-up consultations. Pictures of the last follow-up were taken and retrospectively analyzed by five Board-certified plastic surgeons, resulting in a mean total MSS score of 7.3 (range 4–12; SD ± 2.1). The mean total MSS score of the cases with residual defects requiring further surgical interventions was 8.4 (range 5–12; SD ± 1.8) when compared to 5.9 (range 4–10; SD ± 1.8) of the cases without residual defects. More specifically, the mean MSS score for the category color only was 2.2 (range 1–4; SD ± 0.7) for all patients, of the patients with residual defects 2.5 (range 1–4; SD ± 0.6), and 1.9 (range 1–3; SD ± 0.6) of the cases without residual defects. The mean MSS score for the category finish only was 1.1 (range 1–2; SD ± 0.3) for all patients, of the patients with residual defects 1.2 (range 1–2; SD ± 0.4), and 1.1 (range 1–2; SD ± 0.3) of the cases without residual defects. The mean MSS score for the category contour only was 1.8 (range 1–3; SD ± 0.7) for all patients, of the patients with residual defects 2.1 (range 1–3; SD ± 0.6), and 1.5 (range 1–3; SD ± 0.7) of the cases without residual defects. The mean MSS score for the category distortion was 1.9 (range 1–4; SD ± 0.8) for all patients, of the patients with residual defects 2.3 (range 1–4; SD ± 0.7), and 1.4 (range 1–3; SD ± 0.6) of the cases without residual defects. The mean total MSS score, as well as specifically the categories color, finish, contour, and distortion, were higher for the cases with residual defects when compared to those without residual defects. No clear trend favoring better aesthetic outcomes in either younger patients aged 28–40 (*n* = 3, mean MSS = 7.6) or older patients aged 41–68 (*n* = 6, mean MSS = 7.1) was found. In Figure 2, the MSS scores of the cases with and without residual defects are presented. Pictures of the initial injury, healing process after SVF and PRF application, as well as the final outcome of patients #1, #5, and #11 are demonstrated in Figure 3, Figure 4, Figure 5 and Figure 6 (Table 3).

## 4. Discussion

The eschar debridement followed by STSG coverage still remains the gold standard in the treatment of MPB, DPTB, and FTB. New technologies aim to mitigate the drawbacks of STSG in burn wound treatment. SVF and PRF offer promising, less-invasive alternatives for wound treatment by enhancing epithelialization, angiogenesis, and immunomodulation and have therefore gained a lot of traction amongst researchers recently [9,21,22,23].

The stasis zone surrounding the coagulation zone is an important part determining the irreversible tissue loss in burn injuries. Damage in this area can potentially be salvaged by decreasing the amount of inflammatory mediators released from burned tissue resulting in further cell death due to ischemia [24,25]. Eyuboglu et al. investigated the influence of intradermally injected SVF on the stasis zone of burn wounds in an experimental rat model [26]. SVF was gained enzymatically using collagenase and then injected into the stasis zone after thermal injuries were applied on the dorsum of rats in the form of a comb burn model. The injection of SVF showed a significant increase in vascular density and viable tissue, while the inflammatory cell density was decreased on the 7th day after the burn injury when compared to the control group, which only received a phosphate buffer solution. Compared to our study, the intradermal injection method, as performed in the aforementioned research, delivered SVF directly into the affected area, thereby ensuring a high concentration reached the targeted site. Conversely, the risk of failing to deliver the full healing potential when the SVF is not injected into the precise layer may be mitigated by the topical application form, which provides a more diffuse distribution of the cells. Additionally, locally injected cells are less exposed to external factors such as air and contamination, which could compromise the regenerative properties observed in the topical application method utilized in our study. Nevertheless, the intradermal injection necessitates a high level of skill and is more invasive compared to the topical application form, thereby resulting in an elevated risk of infection.

Further supportive evidence for the regenerative properties of local SVF injection was shown in a clinical case presentation regarding a female patient with 3.5% MPB in the facial area. She received subcutaneous injections with SVF followed by a double-layer collagen dressing resulting in satisfying healing with good cosmetic and functional outcome, especially in the perioral and periorbital areas [27]. The SVF utilized for treatment was obtained using the Stempia^®^ Adipose-Derived Stem Cell Isolation Kit (N-Biotek, Bucheon-si, Republic of Korea), which is based on enzymatic SVF isolation. In our study, patient #2 exhibited residual defects in the facial area. We hypothesize that for facial applications, the injective form of Stromal Vascular Fraction (SVF) may be more effective than topical application. This preference is due to the tendency of dressings to become detached in facial areas, which could inhibit the cells from fully exerting their potential. Therefore, ensuring the cells remain in place via injection could enhance the therapeutic outcomes.

While the injection of SVF appears to be promising, little is known about the efficacy of the topical application of SVF and PRF on the surface of wounds as utilized in our patients.

After enzymatic debridement with Nexobrid^®^, Giudice et al. treated MPB areas of 4% TBSA in a 50-year-old female patient with enzymatically gained SVF distributed over a commercially available Hyalomatrix^®^, a bilayer of a hyaluron scaffold beneath a silicone membrane, or Hyalomatrix^®^ alone [28]. The first dressing change was performed after 15 days, which is 10 days later than the first dressing change in our study. The authors observed a time-to-heal of 25 days in the area treated with SVF/Hyalomatrix^®^, whereas the control group only showed a 23% reduction of the wound surface at this timepoint.

The time-to-heal measured in our study was an average of 20 days for wounds treated with SVF and PRF that healed without residual defects. These findings are consistent with the study conducted by Giudice et al. and appear to indicate a longer healing period compared to the expected time-to-heal for STSGs. The similar healing times shown by Giudice et al. and our study also indicate that the secondary dressing (Hyalomatrix^®^ vs. Mepitel^®^) after SVF application may not be decisive for the outcome. The literature claims the median healing time of burn wounds treated with STSG to be 14 days on average, which is in agreement with the findings in our study, where residual defect coverage with STSG healed within 13.4 days (Table 2) [29].

We assume that the topical application of SVF is even more dependent on a vital wound bed to unfold its true beneficial potential, which highlights the importance of an adequate eschar debridement beforehand. In this context, an insufficient surgical or enzymatical debridement might have been a potential source for the therapy failure of SVF and PRF in our patients. The mode of eschar removal did not predict the outcomes, as residual defects were found within the enzymatically as well as the surgically debrided group. Di Lonardo et al. histologically analyzed burn wounds before and after enzymatical debridement and surgically debrided wounds 24 h after trauma. Their biopsies after enzymatical debridement showed structural alterations affecting the dermal remnants with partial viability. This remaining dermal portion may serve as a natural scaffold to the topically applied SVF and PRF where SVF cells in particular may promptly integrate [30]. Nevertheless, if this dermal scaffold is not managed adequately, particularly with regards to hydration, a neo-eschar might develop resulting in therapy failure. Furthermore, in severely burned patients requiring high volume fluid resuscitation, topically applied SVF and PRF may not properly attach to the wound bed due to the high fluid secretion. These risks may be reduced when SVF and PRF are locally injected and not topically applied. The comparison of the intradermal and topical application of SVF and PRF in burn patients on a larger scale could therefore be of great interest and possibly present a viable adjunct in severely burned patients with limited donor sites.

In our study, we combined the topical application of SVF with PRF. PRF is a valuable concentrate of fibrin and platelets obtained from a centrifugation process of the patient’s blood. It is rich in growth factors and cytokines which aid in accelerating the healing process [31,32]. PRF differs from platelet rich plasma (PRP) as it incorporates a fibrin matrix which imparts a more solid, gel-like consistency that may foster a prolonged and sustained release of growth factors [33], demonstrating promising results in wound treatment [34,35]. In a recent in vivo study, Lektemur et al. investigated the influence of injectable PRF on mucosal wound healing and local vascular endothelial growth factor (VEGF) expression [23]. They created 5 mm wounds in the middle of the buccal mucosa of rats, following five local injections with PRF and saline in the control group. Their results presented an accelerated full epithelialization after 21 days in the PRF group and a significant increase in local VEGF compared to the control group where saline was injected. The timeframe of approximately 21 days until complete epithelialization aligns with the average healing duration identified in a study by Schulz et al. where ten patients with MPB or FTB were treated with topically applied PRF. Similar to the majority of cases in our study, the debridement was performed enzymatically with Nexobrid^®^ and the initial dressing change occurred five days after PRF application. With an average healing time of 18 days, they concluded that the application of PRF enhanced wound healing and reduced the risk of infection; however, 3 out of the 10 patients exhibited residual defects requiring further STSG coverage, which again delineates the limitations of a PRF only or PRF/SVF combination [22].

With the topical application of SVF in combination with PRF, we estimate that PRF will serve as a source of growth factors to boost SVF action, and furthermore, serve as a scaffold promoting better adherence, integration, and survival of the transplanted stromal cells. In a recent in vivo study by Josh et at. the combination of SVF and PRF was locally injected into DPTBs of rats and inhibited the local and systemic oxidative stress response by lowering the levels of malondialdehyde and nitric oxide in blood serum and burned tissue [36]. Laidding et al. investigated the effect of a combination of PRP and SVF on the VEGF serum level in vivo using a rat model with DPTB [37]. They established that the topical and the intradermal application of SVF and PRP resulted in a significant increase in VEGF serum levels compared to the control group. Interestingly, the intradermal injection showed a significantly higher VEGF serum concentration when compared to the topical group, suggesting it to be the more effective route to help increase VEGF serum levels.

In our series, the SVF and PRF processing mechanism and application were safe, and no adverse events were observed. In the last follow-up, all patients exhibited acceptable scar formation; however, patients treated with SVF and PRF without residual defects demonstrated marginally superior outcomes in terms of color, distortion, and contour. Nonetheless, these findings should be interpreted with caution due to the limited sample size, which restricts statistical analysis, and the variability in burn depth and other patient/surgery-related characteristics.

SVF isolation in particular is a time-consuming process. The time for SVF isolation may be reduced by the mechanical processing technique of SVF. SVF presents strong wound-healing properties due to the preservation of the natural extracellular matrix and growth factors [38]. Mechanical processing techniques gain major importance as the enzymatic digestion of adipose tissue with collagenase has been restricted by both the US Food and Drug Administration and the European Medicines Agency (EMA), and also Swissmedic in the recent past, which prohibits the utilization of any enzymatic processing techniques including the GID SVF-2 System [39,40]. This is due to the fact that enzymatic digestion is considered a form of “more-than-minimal manipulation” under regulatory frameworks, as it alters the original relevant biological characteristics or the structural integrity of the tissue or cells. According to current regulatory definitions, such manipulation exceeds the threshold of minimal manipulation because it may impact the tissue’s intended function or use. Furthermore, the integration of SVF and PRF into clinical care requires additional procedural time, resources, and costs beyond those of standard treatment. This may restrict its applicability, particularly in resource-limited settings. In addition, the requirement for specialized equipment and trained personnel for both adipose tissue harvesting and autologous blood processing introduces practical and logistical challenges.

The weaknesses of this study are the heterogeneity of the patients of a non-powered sample size. Due to the mentioned national regulatory restrictions, the use of enzymatically processed SVF is currently no longer permitted, which precludes the inclusion of additional cases at this time. The approach presented here is expensive and labor-intensive; therefore, its use is confined to specialized facilities with ample resources, such as the Burn Center in Zurich, which faces challenges in obtaining a larger patient cohort. Despite the limited sample size, we believe our findings provide valuable clinical insights and contribute to the expanding literature on regenerative strategies in burn care. Additionally, the heterogeneity of mixed-pattern burns, which were frequently observed among our patients, presents an additional challenge in defining clear indications for the use of SVF and PRF. This variability not only complicates the therapeutic decision-making process but also limits the ability to reliably assess and compare wound-healing outcomes across cases. We also acknowledge the wide range of total body surface area (TBSA) involvement in our study (6–85%) as a limitation. While this spectrum reflects the clinical reality and diversity of burn severity encountered in everyday practice, it likely contributes to variability in treatment outcomes. Moreover, the potential impact of burn shock on the quality of adipose tissue used for SVF harvesting remains insufficiently understood. Systemic inflammation, ischemia, and reperfusion injury associated with the early post-burn phase may theoretically impair the viability and regenerative capacity of adipose-derived cells. However, based on our clinical experience, we did not observe any overt compromise in the macroscopic quality of SVF when liposuction was performed shortly after initial fluid resuscitation. Nonetheless, further research is needed to evaluate whether functional alterations in SVF may occur under such conditions.

## 5. Conclusions

Taken together, the topical application of SVF and PRF permits an alternative, lightly invasive therapy option for acute thermal injuries that is backed by promising preclinical data. Due to residual defects requiring further surgical interventions in almost 45% of our patients, we conclude that the topical application of SVF and PRF does not present a superior option compared to standard skin grafting at this stage. Nevertheless, this study shows that complete wound healing can be achieved with an acceptable cosmetic outcome and few side effects. Furthermore, SVF and PRF may present a useful adjunct in severely burned patients with limited donor sites and possibly help reduce the total wound surface. However, an optimization of treatment protocols including mechanical SVF isolation protocols may be required to improve outcomes and bypass existing regulatory boundaries in most countries.

## Figures and Tables

**Table 1:** Patient characteristics regarding age, ABSI score, TBSA, BSA treated with SVF and PRF, and time from trauma to debridement as well as time from initial debridement to SVF and PRF application.

Variable	Mean	SD
Age [years]	45.3	13.5
ABSI score	6.5	3.5
Total body surface area (TBSA) [%]	29.6	25.8
Body surface area (BSA) treated with SVF and PRF [%]	6.3	4.7
Time from trauma to initial debridement [days]	1.6	0.7
Time from initial debridement to SVF and PRF application [days]	2.4	2.1

**Table 2:** Specification of areas with residual defects requiring further surgery and time-to-heal.

Variable	Mean	SD
Patients with residual defects requiring further surgery [%]	45	-
BSA residual defects [%]	6.6	6
BSA residual defects/BSA-treated area in patients with residual defects [%]	91	-
Time-to-heal all patients [d]	33	17.7
Time-to-heal with residual defects [d]	51	8.8
Time-to-heal without residual defects [d]	20	7.5
Time-to-heal after residual defect coverage with STSG [d]	13.4	4.6

**Table 3:** Characteristics of all 13 patients treated with SVF and PRF.

Patient	Age	Sex	Comorbidities	Burn Etiology	BSA	ABSI Score	A: BSA Treated with SVF and PRF B: Localization C: Burn Degree	Debridement of Area Treated with SVF and PRF	Time Trauma to Initial Debridement	Time Initial Debridement to SVF and PRF	Treatment	BSA of Residual Defects	Time SVF and PRF to Treatment of Residual Defects	Treatment of Residual Defects	Time-to-Heal	Hospitalization Time A: Total B: ICU C: Normal Ward	Follow-Up	Manchester Scar Score
1	41	M	-	fire	Total: 65% SPTB: 15% DPTB: 46% FTB: 4%	12	A: 3% B: forearms/hands C: DPTB	enzymatic	2 d	2 d	SVF + PRF	3%	14 d	STSG	57 d	A: 111 d B: 76 d C: 35 d	66 m	6
2	31	F	Polytoxicomania	fire	Total: 36% SPTB: 2% DPTB: 11% FTB: 23%	8	A: 3% B: head C: SPTB/DPTB	manual (tangential)	3 d	same day	SVF + PRF	2%	27 d	STSG	60 d	A: 73 d B: 50 d C: 23 d	30 m	10
3	35	M	Hypertonia	fire	Total: 26% SPTB: 5% DPTB: 10% FTB: 11%	6	A: 10% B: anterior torso C: DPTB/FTB	manual (epifascial)	2 d	same day	SVF + PRF + STSG	-	-	-	28 d	A: 39 d B: 29 d C: 10 d	n/a	n/a
4	68	M	Steatosis Hepatis	fire	Total: 18% SPTB: 16% DPTB: 2%	6	A: 4% B: hands C: DPTB	enzymatic	1 d	6 d	SVF + PRF	-	-	-	25 d	A: 37 d B: 15 d C: 22 d	15 m	9
5	42	M	-	fire	Total: 11.5% SPTB: 6.5% DPTB: 5%	4	A: 3% B: hand/upper arm C: SPTB/DPTB	enzymatic	1 d	6 d	SVF + PRF	-	-	-	14 d	A: 16 d B: 0 d C: 16 d	60 m	4
6	52	W	-	fire	Total: 17% SPTB: 2.5% DPTB: 5% FTB: 2.5%	7	A: 1% B: dorsal hand C: SPTB/DPTB	enzymatic	1 d	2 d	SVF + PRF	-	-	-	20 d	A: 25 d B: 5 d C: 20 d	n/a	n/a
7	51	W	-	scald	Total: 13% SPTB: 2% DPTB: 11%	5	A: 11% B: gluteal area C: DPTB	enzymatic	1 d	4 d	SVF + PRF + STSG	11%	17 d	MEEK	40 d	A: 46 d B: 33 d C: 13 d	24 m	8
8	54	M	-	fire	Total: 11% SPTB: 4% DPTB: 7%	3	A: 3% B: hands/forearm C: SPTB/DPBT	enzymatic	1 d	2 d	SVF + PRF	1%	34 d	STSG	50 d	A: 21 d B: 3 d C: 18 d	24 m	10
9	55	W	Psoriasis	scald	Total: 6% SPTB: 4% DPTB: 2%	3	A: 3% B: hands, foot, neck C: SPTB/DPTB	enzymatic	1 d	3 d	SVF + PRF	-	-	-	14 d	A: 10 d B: 0 d C: 10 d	24 m	6
10	35	M	Polytoxicomania	scald	Total: 15% SPTB: 7% DPTB: 8%	4	A: 15% B: right leg, abdomen, forearm C. SPTB/DPTB	manual (tangential)	3 d	same day	SVF + PRF	15%	11 d	STSG	n/a	A: 31 d B: 2 d C: 29 d	45 m	8
11	28	W	-	scald	Total 15% SPTB: 10% DPTB: 5%	3	A: 15% B: thighs, abdomen C: SPTB/DPTB	enzymatic	1 d	4 d	SVF + PRF	-	-	-	28 d	A: 12 d B: 0 d C: 12 d	30 m	5
12	30	W	Depression	fire	Total: 85% SPTB: 10% DPTB: 25% FTB 50%	13	A: 3% B: face C: DPTB	enzymatic	2 d	3 d	SVF + PRF	n/a	n/a	n/a	n/a	Death after 11 d	n/a	n/a
13	67	M	-	fire	Total: 65% DPTB: 35% FTB: 30%	11	A: 4% B: right arm C: DPTB	manual (tangential)	2 d	same day	SVF + PRF + STSG	n/a	n/a	n/a	n/a	Death after 19 d	n/a	n/a

M: male; F: female; BSA: body surface area; SPTB: superficial partial-thickness burn; DPTB: deep partial-thickness burn; FTB: full-thickness burn; STSG: split-thickness skin graft; SVF: stromal vascular fraction; PRF: platelet-rich fibrin; ABSI: abbreviated burn severity index; ICU: intensive care unit; d: day; m: month; n/a: not available.

## Data Availability

The datasets used and/or analyzed in this study are available from the corresponding author on reasonable request.
